# Genetic diversity of *Pantoea stewartii* subspecies *stewartii* causing jackfruit-bronzing disease in Malaysia

**DOI:** 10.1371/journal.pone.0234350

**Published:** 2020-06-12

**Authors:** Nuraizat Abidin, Siti Izera Ismail, Ganesan Vadamalai, Mohd Termizi Yusof, Mansor Hakiman, Daljit Singh Karam, Noor Wahida Ismail-Suhaimy, Rohaya Ibrahim, Dzarifah Zulperi

**Affiliations:** 1 Department of Plant Protection, Faculty of Agriculture, Universiti Putra Malaysia, Serdang, Selangor, Malaysia; 2 Department of Microbiology, Faculty of Biotechnology and Biomolecular Sciences, Universiti Putra Malaysia, Serdang, Selangor, Malaysia; 3 Department of Crop Science, Faculty of Agriculture, Universiti Putra Malaysia, Serdang, Selangor, Malaysia; 4 Department of Land Management, Faculty of Agriculture, Universiti Putra Malaysia, Serdang, Selangor, Malaysia; 5 Laboratory of Sustainable Resources Management, Institute of Tropical Forestry and Forest Products, Universiti Putra Malaysia, Serdang, Selangor, Malaysia; Deen Dayal Upadhyaya Gorakhpur University, INDIA

## Abstract

Jackfruit-bronzing is caused by bacteria *Pantoea stewartii* subspecies *stewartii* (*P*. *stewartii* subsp. *stewartii*), showing symptoms of yellowish-orange to reddish discolouration and rusty specks on pulps and rags of jackfruit. Twenty-eight pure bacterial strains were collected from four different jackfruit outbreak collection areas in Peninsular Malaysia (Jenderam, Maran, Muadzam Shah and Ipoh). Positive *P*. *stewartii* subsp. *stewartii* verification obtained in the study was based on the phenotypic, hypersensitivity, pathogenicity and molecular tests. Multilocus sequence analysis (MLSA) was performed using four housekeeping genes (*gyr*B, *rpo*B, *atp*D and *inf*B) on all 28 bacterial strains. Single *gyr*B, *rpo*B, *atp*D and i*nf*B phylogenetic trees analyses revealed the bootstrap value of 99–100% between our bacterial strains with *P*. *stewartii* subsp. *stewartii* reference strains and *P*. *stewartii* subsp. *indologenes* reference strains. On the other hand, phylogenetic tree of the concatenated sequences of the four housekeeping genes revealed that our 28 bacterial strains were more closely related *to P*. *stewartii* subsp. *stewartii* (99% similarities) compared to its close relative *P*. *stewartii* subsp. *indologenes*, although sequence similarity between these two subspecies were up to 100%. All the strains collected from the four collection areas clustered together, pointing to no variation among the bacterial strains. This study improves our understanding and provided new insight on the genetic diversity of *P*. *stewartii* subsp. *stewartii* associated with jackfruit-bronzing in Malaysia.

## Introduction

Jackfruit *(Artocarpus heterophyllus*) comes from the mulberry family (Moraceae) and is believed to originate from the evergreen forest in Western Ghast, India [1]. Jackfruit is an important crop in Malaysian agriculture and recognised as “Nangka” by Malay verbal. The Economic Transfer programme (ETP) implemented by the Government of Malaysia [2] that includes National Key Economic Area (NKEA) states that “Jackfruit is an important driver of economic activities that potentially and directly contributes toward the Malaysia economic growth measurable by the National Gross Income (GNI) indicator” [3]. Under NKEA Agriculture, which includes Entry Point Project number 7 (EPP 7), it focuses on the increased growth of premium fruits and vegetables of which jackfruit is one of the six high-value fruit crops of EPP 7 [4].

Jackfruit industry in Malaysia is currently under threat with a disease named “jackfruit-bronzing”. Examining and keeping inspection records on the status of jackfruit-bronzing is essential to preserve a high quality and to ensure the security of the local food supply of jackfruit. Recently, there has been three reported cases of jackfruit-bronzing in the Philippines [5], Peninsular Malaysia [6] and Mexico [7]. Visually, bronzing disease on jackfruit is symptomless on the external appearance of the fruit, but it shows yellowish-orange to reddish discolouration and rusty specks on the pulps and rags [5–7]. The other visible symptom according to an earlier report on the outbreak of the disease in Malaysia was obvious reddish-brown spots on the pulps [8].

The causal agent, *P*. *stewartii* subsp. *stewartii* (Smith) Mergaert is a plant pathogenic bacterium of the family *Enterobacteriaceae* [9]. This bacteria is the only pathogen listed in the quarantine pathogen among the genus *Pantoea* [10]. The European and Mediterranean Plant Protection Organization (EPPO) stated maize as the primary host for *P*. *stewartii* subsp. *stewartii*, which is widely known as Stewart’s bacterial wilt as well as leaf blight of corn, sweetcorn, maize, dent, flint, flour and popcorn plant, indigenous to North America [11,12]. The first case of a plant disease ever reported among the genus *Pantoea* was Stewart’s wilt of maize, and the discovery led to major losses in crop yield [13,14].

*P*. *stewartii* subsp. *stewartii* can be identified using the primers designed from the 16S-23S ribosomal DNA (rDNA) internal transcribed spacer (ITS) [6,14] to confirm the identity of the bacterial strains. As the pure bacterial strains have been confirmed positive for *P*. *stewartii* subsp. *stewartii*, pathogenicity test is vital to confirm if the bacterium is the causal agent, or this would disprove their status as novel pathogens [9,15]. Multilocus sequence analysis (MLSA) is considered as the novel standard for the systematics in molecular microbiology expected to produce high resolving power results [16]. Based on partial sequence of *gyr*B (encoding DNA gyrase subunit B), *rpo*B (encoding RNA polymerase beta subunit), *atp*D (encoding ATP synthase F1, β-subunit) and *inf*B (encoding Translation initiation factor IF-2) genes (also known as Housekeeping genes), *P*. *stewartii* subsp. *stewartii* is successfully distinguished from another *Pantoea* genus. So far, the genetic diversity of *P*. *stewartii* subsp. *stewartii* in Peninsular Malaysia has not been investigated.

Losses in jackfruit yields began with continuous fruit bronzing disease occurrences [6]. It leads to loss of jackfruit production and thus considered as a major constrain with big economic impact in Malaysia [6]. Since the first outbreak in 2017, very limited documentations of jackfruit-bronzing disease in Malaysia has been recorded. Hence, detailed status reports of jackfruit-bronzing disease is significantly important since jackfruit remains as one of the most important commercial fruit crops with a high economic value in Malaysia.

In connection with the first report of *P*. *stewartii* subsp. *stewartii* infected jackfruit in 2017 in Muadzam Shah, Peninsular Malaysia, the objectives of the present study were: (i) to verify the bacterial strains associated with bronzing disease of jackfruit in four outbreak plantations in Peninsular Malaysia are *P*. *stewartii* subsp. *stewartii*, using phenotypic and molecular characteristics; and (ii) to elucidate the genetic diversity of *P*. *stewartii* subsp. *stewartii* strains associated with bronzing disease of jackfruit using multilocus sequence analysis (MLSA) approach.

## Materials and methods

### Sample collection and isolation of bacterial pathogen

A total of 28 diseased jackfruits (Tekam Yellow J33 variety) were collected from outbreak plantation areas in Peninsular Malaysia, comprising of 7 fruits from Jenderam (Selangor), 7 fruits from Maran (Pahang), 7 fruits from Muadzam Shah (Pahang) and 7 fruits from Ipoh (Perak) from September-November 2017 (Table 1). Fruits were collected randomly, with three replicates for each sample per location (total 21 fruits per collection area). The infected fruits were wrapped, put inside sterile plastic bags, labelled and brought to the laboratory for initial diagnosis and isolation of the pathogen. The isolation procedure was conducted not later than 24 h after the collected samples were brought to the laboratory.

**Table 1:** Sources of isolation, collection area and jackfruit variety of 28 *Pantoea stewartii subspecies stewartii* strains in Peninsular Malaysia used in this study.

NI^1^	ID^2^	Collection area	State	Host	Jackfruit variety
1	JEN-3	Jenderam	Selangor	*Artocarpus heterophyllus*	Tekam Yellow (J33)
2	JEN-5	Jenderam	Selangor	*Artocarpus heterophyllus*	Tekam Yellow (J33)
3	JEN-8	Jenderam	Selangor	*Artocarpus heterophyllus*	Tekam Yellow (J33)
4	JEN-13	Jenderam	Selangor	*Artocarpus heterophyllus*	Tekam Yellow (J33)
5	JEN-14	Jenderam	Selangor	*Artocarpus heterophyllus*	Tekam Yellow (J33)
6	JEN-16	Jenderam	Selangor	*Artocarpus heterophyllus*	Tekam Yellow (J33)
7	JEN-20	Jenderam	Selangor	*Artocarpus heterophyllus*	Tekam Yellow (J33)
8	MAR-A	Maran	Pahang	*Artocarpus heterophyllus*	Tekam Yellow (J33)
9	MAR-D	Maran	Pahang	*Artocarpus heterophyllus*	Tekam Yellow (J33)
10	MAR-E	Maran	Pahang	*Artocarpus heterophyllus*	Tekam Yellow (J33)
11	MAR-F	Maran	Pahang	*Artocarpus heterophyllus*	Tekam Yellow (J33)
12	MAR-H	Maran	Pahang	*Artocarpus heterophyllus*	Tekam Yellow (J33)
13	MAR-M	Maran	Pahang	*Artocarpus heterophyllus*	Tekam Yellow (J33)
14	MAR-Q	Maran	Pahang	*Artocarpus heterophyllus*	Tekam Yellow (J33)
15	MS-3	Muadzam Shah	Pahang	*Artocarpus heterophyllus*	Tekam Yellow (J33)
16	MS-4	Muadzam Shah	Pahang	*Artocarpus heterophyllus*	Tekam Yellow (J33)
17	MS-8	Muadzam Shah	Pahang	*Artocarpus heterophyllus*	Tekam Yellow (J33)
18	MS-B	Muadzam Shah	Pahang	*Artocarpus heterophyllus*	Tekam Yellow (J33)
19	MS-C	Muadzam Shah	Pahang	*Artocarpus heterophyllus*	Tekam Yellow (J33)
20	MS-F	Muadzam Shah	Pahang	*Artocarpus heterophyllus*	Tekam Yellow (J33)
21	MS-H	Muadzam Shah	Pahang	*Artocarpus heterophyllus*	Tekam Yellow (J33)
22	IPOH-5	Ipoh	Perak	*Artocarpus heterophyllus*	Tekam Yellow (J33)
23	IPOH-B	Ipoh	Perak	*Artocarpus heterophyllus*	Tekam Yellow (J33)
24	IPOH-I	Ipoh	Perak	*Artocarpus heterophyllus*	Tekam Yellow (J33)
25	IPOH-M	Ipoh	Perak	*Artocarpus heterophyllus*	Tekam Yellow (J33)
26	IPOH-S	Ipoh	Perak	*Artocarpus heterophyllus*	Tekam Yellow (J33)
27	IPOH-V	Ipoh	Perak	*Artocarpus heterophyllus*	Tekam Yellow (J33)
28	IPOH-Z	Ipoh	Perak	*Artocarpus heterophyllus*	Tekam Yellow (J33)

Abbreviation: NI^1^ = Number of strains; ID^2^ = Strains designation.

Infected samples were excised, cut and rinsed with sterilized distilled water (containing 1% of sodium hypochlorite, Chlorox™) for 3 minutes. The jackfruit pulps were put on King’s B agar medium, sealed properly and incubated in inverted position for 24 to 48 h at 28º C. After incubation, isolated colonies were subcultured until pure colonies of suspected *P*. *stewartii* subsp. *stewartii* strains were obtained. As mentioned by EPPO, the colonies were expected to be lemon to pale-yellow or orange-yellow in colour, flat to convex, transparent with entire edges, and slow to medium growing [9]. For later use, the pure bacterial cultures were grown in nutrient broth medium with 20% (v/v) glycerol and stored at -80°C.

### Ethics statement

For collection of fruits, no specific permits or permission were required for these locations/activities. All field research was conducted on privately-owned farms and with owners’ permission. All locations where the fruits were collected did not involve endangered or protected species.

### Verification via phenotypic characterization

The positive phenotypic characterization for the verification of *P*. *stewartii* subsp. *stewartii* were performed based on previous studies verification [5,9,17–19] as mentioned on S1 Table. All tests were performed using the freshly grown colonies of *P*. *stewartii* subsp. *stewartii* (24 to 48 h).

### Verification via molecular characterization

*P*. *stewartii* subsp. *stewartii* strains were grown on nutrient broth for 24 to 48 h at 28°C. Using commercial genomic DNA isolation kit, (Presto^TM^ Mini gDNA Bacteria Kit, Geneaid Biotech LTD., Taiwan), all the 28 bacteria strains were extracted following the protocol provided. The polymerase chain reaction (PCR) was performed in a 25 μl reaction mixture, contained 1 μg of extracted total genomic DNA template, 12.5 μl of 2x DreamTaq Red PCR MasterMix (Thermo Scientific Inc., USA), 9.5 μl of DNase-free water and 1 μl of each primer (Apical Scientific, Malaysia). PCR amplification was performed following protocols, in ‘iCycler’ Thermal Cycler (Bio-Rad Laboratories Inc., USA) to amplify the 16S-23S ITS region, using primer pair: ES16 (5’- GCG AAC TTG GCA GAG AT -3’) and ESIG2c (5’- GCG CTT GCG TGT TAT GAG -3’) [14,20].

### Hypersensitive assay

Four-week-old *Nicotiana benthamiana* transplants were used for hypersensitivity assays. Using syringe, 1 ml of 10^8^ CFU/ml of bacterial suspension (as determined by McFarland Standard, BioMérieux, Marcyl'Etoile, France) was inoculated into a fully expanded tobacco leaf (3 leaves per bacterial culture). Leaves infiltrated with sterile distilled water served as a negative control. The greenhouse condition maintained at temperature range from 26°C to 35°C. The plant response for Hypersensitivity reaction (HR) was recorded after 10 to 36 h post-infiltration. Necrosis of the infiltrated tissues was considered as positive HR, and the absence was considered as a negative test. Four replicates were performed for each strain due to any imprecision concern. Two results were expected for this test, as past studies showed *P*. *stewartii* subsp. *stewartii* strains resulted in positive HR and pathogenic [18] or otherwise resulting negative HR, suggesting that the strains are non-pathogenic due to mutation in the *hrp* gene [5,11,18].

### Pathogenicity test

Detached healthy jackfruit pulps (Tekam Yellow J33 variety) were inoculated with 10 mL of 10^8^ CFU/mL (as determined by McFarland Standard, BioMérieux, Marcyl'Etoile, France) of 24 to 48 h pure *P*. *stewartii* subsp. *stewartii* suspensions. Sterile distilled water was inoculated to control healthy jackfruit pulps. 4 replicates were performed per strain. All 28 bacterial strains treatments were put inside sterile petri dish (properly sealed) and incubated at 28 ºC in a controlled chamber. The evaluation of jackfruit-bronzing symptoms was recorded daily up to two weeks of inoculation.

### MLSA

All extracted total genomic DNA were used as a template in the PCR amplification using primers as listed on S2 and S3 Tables. The PCR reactions were performed as described under the previous section.

### Electrophoresis of PCR products, DNA purification, sequencing, sequence alignment, phylogenetic analyses and dendrogram construction

Separation of PCR products was performed on a prepared 1% agarose gel with FloroSafe DNA stain. Using Mini Sub™ DNA Cell, (Bio-Rad Laboratories Inc., USA), the PCR amplicons was run at 75 V (Power supply model 1000/500, Bio-Rad Laboratories Inc., USA) for 45 min and later visualized and photographed by Alpha Imager System (Alpha-Innotech, Siber-Hegner, UK).

All amplified PCR products were then sent for purification and sequencing (MyTACG Sequencing, Malaysia). All forward and reverse sequences were assembled and analyzed using Bioedit version 7.2.5 [21]. The FASTA format of the gene sequences were subjected to BLASTn (http://blast.st-va.ncbi.nlm.nih.gov/Blast.cgi) search to determine their similarity to nucleotide sequences available in the GenBank database. This was followed by using ClustalW multiple alignment program (http://www.ebi.ac.uk/Tools/msa/clustalw2/), with manual adjustments. MEGA7 software [22] was used for the construction of phylogenetic trees using the best fit substitution model identified and calculated by the "Find Best Models" tool. Maximum likelihood tree were then generated with bootstraps values of 1000 for each analysis. The available sequences for these genes in genomes of *P*. *stewartii* subsp. *stewartii* were then compared.

All reference strains were obtained from NCBI online database (S4 and S5 Tables). The sequenced genes from the study have been deposited into the NCBI database as mention on S4 and S5 Tables.

## Results

### Collection of the sample and symptoms of jackfruit-bronzing

**Fig 1:**
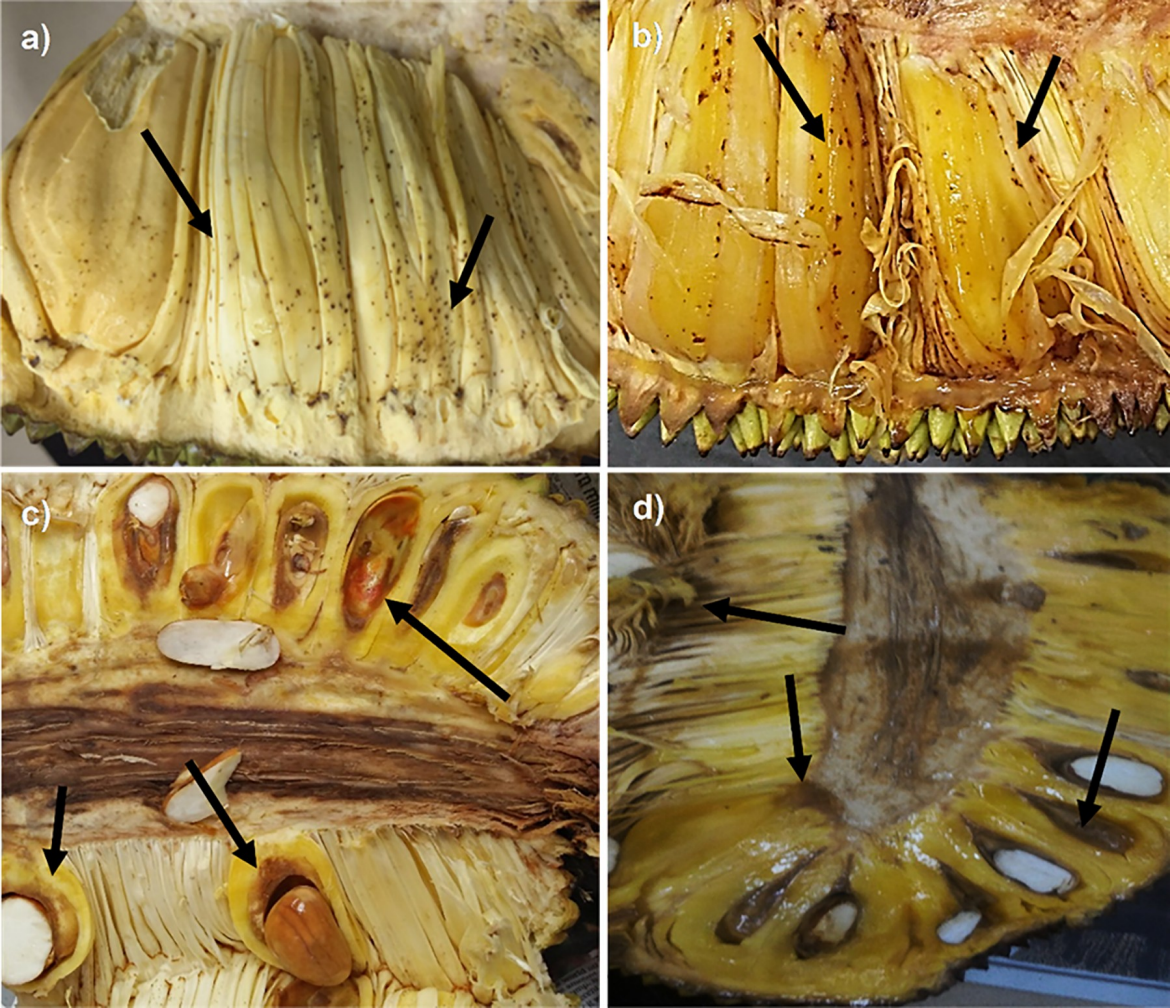
Infected jackfruits showing the symptom of bronzing disease. (a) Yellowish discolouration with bronzing specks symptom collected from Muadzam Shah; (b) Yellowish discolouration with bronzing specks symptom collected from Ipoh; (c) Reddish discolouration on the pulps of jackfruit collected from Jenderam; (d) Reddish discolouration on the pulps of jackfruit collected from Maran.

From the total of 28 collected jackfruits, all fruits (7 fruits from Jenderam, 7 fruits from Maran, 7 fruits from Muadzam Shah and 7 fruits from Ipoh) showed the symptoms of bronzing disease (Table 2). The outer appearance of the collected fruits were symptomless. Internally, they showed visible jackfruit-bronzing symptoms either yellowish discolouration with bronzing specks or reddish discolouration on the pulps and rags (Fig 1). The symptoms were inconsistent with 17 of the fruits showed yellowish discolouration with bronzning specks and 11 had reddish discolouration appearance (Table 2).

**Table 2:** Symptoms and severity level of the collected jackfruit from the four infected collection areas.

NI^1^	ID^2^	Collection area	State	Host	Variety	Symptom
1	JEN-3	Jenderam	Selangor	*Artocarpus heterophyllus*	Tekam Yellow (J33)	R^3^
2	JEN-5	Jenderam	Selangor	*Artocarpus heterophyllus*	Tekam Yellow (J33)	YB^4^
3	JEN-8	Jenderam	Selangor	*Artocarpus heterophyllus*	Tekam Yellow (J33)	R
4	JEN-13	Jenderam	Selangor	*Artocarpus heterophyllus*	Tekam Yellow (J33)	YB
5	JEN-14	Jenderam	Selangor	*Artocarpus heterophyllus*	Tekam Yellow (J33)	YB
6	JEN-16	Jenderam	Selangor	*Artocarpus heterophyllus*	Tekam Yellow (J33)	YB
7	JEN-20	Jenderam	Selangor	*Artocarpus heterophyllus*	Tekam Yellow (J33)	R
8	MAR-A	Maran	Pahang	*Artocarpus heterophyllus*	Tekam Yellow (J33)	YB
9	MAR-D	Maran	Pahang	*Artocarpus heterophyllus*	Tekam Yellow (J33)	R
10	MAR-E	Maran	Pahang	*Artocarpus heterophyllus*	Tekam Yellow (J33)	YB
11	MAR-F	Maran	Pahang	*Artocarpus heterophyllus*	Tekam Yellow (J33)	YB
12	MAR-H	Maran	Pahang	*Artocarpus heterophyllus*	Tekam Yellow (J33)	R
13	MAR-M	Maran	Pahang	*Artocarpus heterophyllus*	Tekam Yellow (J33)	YB
14	MAR-Q	Maran	Pahang	*Artocarpus heterophyllus*	Tekam Yellow (J33)	YB
15	MS-3	Muadzam Shah	Pahang	*Artocarpus heterophyllus*	Tekam Yellow (J33)	R
16	MS-4	Muadzam Shah	Pahang	*Artocarpus heterophyllus*	Tekam Yellow (J33)	YB
17	MS-8	Muadzam Shah	Pahang	*Artocarpus heterophyllus*	Tekam Yellow (J33)	YB
18	MS-B	Muadzam Shah	Pahang	*Artocarpus heterophyllus*	Tekam Yellow (J33)	R
19	MS-C	Muadzam Shah	Pahang	*Artocarpus heterophyllus*	Tekam Yellow (J33)	YB
20	MS-F	Muadzam Shah	Pahang	*Artocarpus heterophyllus*	Tekam Yellow (J33)	YB
21	MS-H	Muadzam Shah	Pahang	*Artocarpus heterophyllus*	Tekam Yellow (J33)	YB
22	IPOH-5	Ipoh	Perak	*Artocarpus heterophyllus*	Tekam Yellow (J33)	YB
23	IPOH-B	Ipoh	Perak	*Artocarpus heterophyllus*	Tekam Yellow (J33)	R
24	IPOH-I	Ipoh	Perak	*Artocarpus heterophyllus*	Tekam Yellow (J33)	YB
25	IPOH-M	Ipoh	Perak	*Artocarpus heterophyllus*	Tekam Yellow (J33)	YB
26	IPOH-S	Ipoh	Perak	*Artocarpus heterophyllus*	Tekam Yellow (J33)	YB
27	IPOH-V	Ipoh	Perak	*Artocarpus heterophyllus*	Tekam Yellow (J33)	R
28	IPOH-Z	Ipoh	Perak	*Artocarpus heterophyllus*	Tekam Yellow (J33)	R

Abbreviation: NI^1^ = Number of strains; ID^2^ = Strains designation; R^3^ = Reddish discolouration; YB^4^ = Yellowish discolouration with bronzing specs.

### Phenotypic characterization and molecular characterization

**Fig 2:**
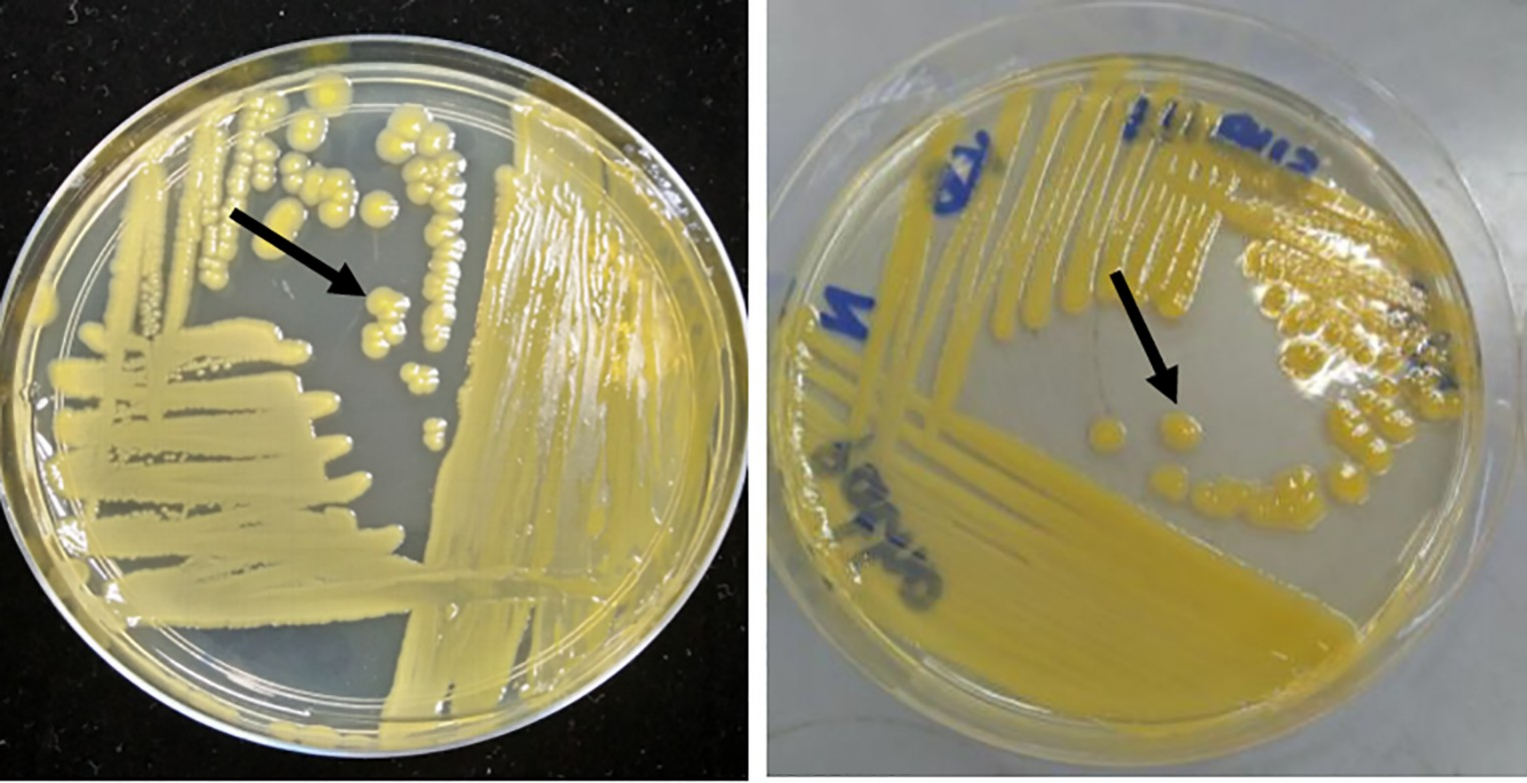
Colony morphology of *Pantoea stewartii* subspecies *stewartii* isolated from infected sampling areas across Peninsular Malaysia on a King’s B agar medium.

**Fig 3:**
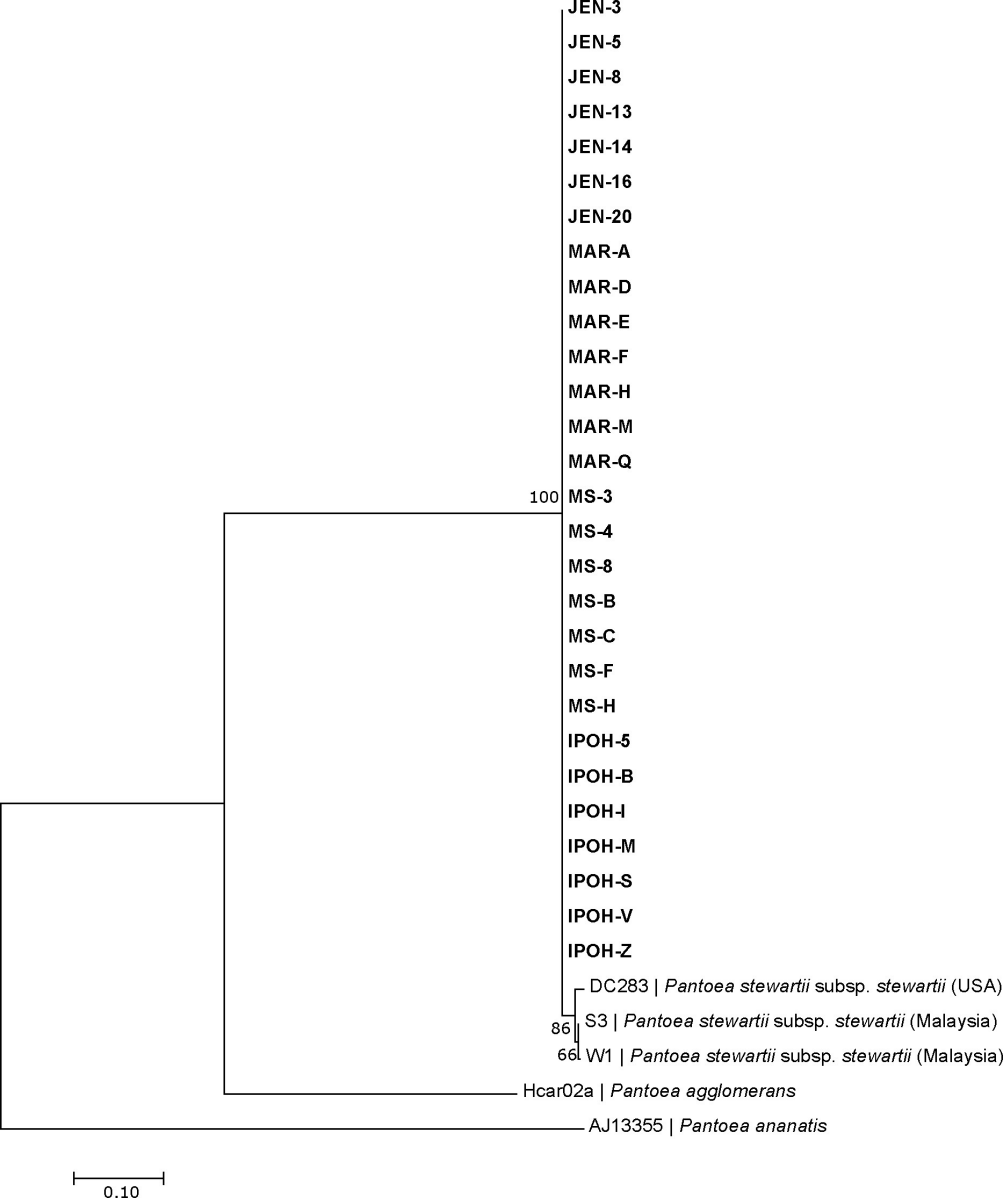
Maximum likelihood tree based on 16S-23S Internally Transcribed Spacer (ITS) region. Bootstrap values after 1000 replicates are expressed as percentages. *Pantoea agglomerans* and *Pantoea ananatis* were included as an outgroup. The scale bar indicates the fraction of substitutions per site.

All the colonies were identified accordingly to the morphology characteristics as mentioned by EPPO [9]. Colonies were lemon-yellow in colour, circular, raised and convex on King’s B agar medium (Fig 2). From the 28 strains, all of the strains were positive and verified for *P*. *stewartii* subsp. *stewartii* identification (Table 3). PCR amplification of all 28 isolated strains using 16S-23S ITS primer pair, each produced a ~900 bp amplicon (S1 Fig). Phylogenetic analysis based on partial 16S-23S ITS gene (Fig 3) revealed all strains isolated in this study were 100% clustered together with the *P*. *stewartii* subsp. *stewartii* reference strains DC283, S3 and W1. None of the strains clustered with *P*. *agglomerans* (*Pantoea agglomerans*) and *P*. *ananatis* (*Pantoea anantis*) strains.

**Table 3:** Phenotypic characterization on 28 strains of *Pantoea stewartii* subspecies *stewartii* from Peninsular Malaysia.

NI^1^	ID^2^	GS^3^	KOH^4^	CR^5^	OR^6^	IP^7^	M^8^	SH^9^	T80^10^	GL^11^	PT^12^	CT^13^			
												G^14^	S^15^	F^16^	L^17^
1	JEN-3	**-**	**+**	**+**	**-**	**-**	**-**	**+**	**-**	**+**	**+**	**+**	**+**	**+**	**+**
2	JEN-5	**-**	**+**	**+**	**-**	**-**	**-**	**+**	**-**	**+**	**+**	**+**	**+**	**+**	**+**
3	JEN-8	**-**	**+**	**+**	**-**	**-**	**-**	**+**	**-**	**+**	**+**	**+**	**+**	**+**	**+**
4	JEN-13	**-**	**+**	**+**	**-**	**-**	**-**	**+**	**-**	**+**	**+**	**+**	**+**	**+**	**+**
5	JEN-14	**-**	**+**	**+**	**-**	**-**	**-**	**+**	**-**	**+**	**+**	**+**	**+**	**+**	**+**
6	JEN-16	**-**	**+**	**+**	**-**	**-**	**-**	**+**	**-**	**+**	**+**	**+**	**+**	**+**	**+**
7	JEN-20	**-**	**+**	**+**	**-**	**-**	**-**	**+**	**-**	**+**	**+**	**+**	**+**	**+**	**+**
8	MAR-A	**-**	**+**	**+**	**-**	**-**	**-**	**+**	**-**	**+**	**+**	**+**	**+**	**+**	**+**
9	MAR-D	**-**	**+**	**+**	**-**	**-**	**-**	**+**	**-**	**+**	**+**	**+**	**+**	**+**	**+**
10	MAR-E	**-**	**+**	**+**	**-**	**-**	**-**	**+**	**-**	**+**	**+**	**+**	**+**	**+**	**+**
11	MAR-F	**-**	**+**	**+**	**-**	**-**	**-**	**+**	**-**	**+**	**+**	**+**	**+**	**+**	**+**
12	MAR-H	**-**	**+**	**+**	**-**	**-**	**-**	**+**	**-**	**+**	**+**	**+**	**+**	**+**	**+**
13	MAR-M	**-**	**+**	**+**	**-**	**-**	**-**	**+**	**-**	**+**	**+**	**+**	**+**	**+**	**+**
14	MAR-Q	**-**	**+**	**+**	**-**	**-**	**-**	**+**	**-**	**+**	**+**	**+**	**+**	**+**	**+**
15	MS-3	**-**	**+**	**+**	**-**	**-**	**-**	**+**	**-**	**+**	**+**	**+**	**+**	**+**	**+**
16	MS-4	**-**	**+**	**+**	**-**	**-**	**-**	**+**	**-**	**+**	**+**	**+**	**+**	**+**	**+**
17	MS-8	**-**	**+**	**+**	**-**	**-**	**-**	**+**	**-**	**+**	**+**	**+**	**+**	**+**	**+**
18	MS-B	**-**	**+**	**+**	**-**	**-**	**-**	**+**	**-**	**+**	**+**	**+**	**+**	**+**	**+**
19	MS-C	**-**	**+**	**+**	**-**	**-**	**-**	**+**	**-**	**+**	**+**	**+**	**+**	**+**	**+**
20	MS-F	**-**	**+**	**+**	**-**	**-**	**-**	**+**	**-**	**+**	**+**	**+**	**+**	**+**	**+**
21	MS-H	**-**	**+**	**+**	**-**	**-**	**-**	**+**	**-**	**+**	**+**	**+**	**+**	**+**	**+**
22	IPOH-5	**-**	**+**	**+**	**-**	**-**	**-**	**+**	**-**	**+**	**+**	**+**	**+**	**+**	**+**
23	IPOH-B	**-**	**+**	**+**	**-**	**-**	**-**	**+**	**-**	**+**	**+**	**+**	**+**	**+**	**+**
24	IPOH-I	**-**	**+**	**+**	**-**	**-**	**-**	**+**	**-**	**+**	**+**	**+**	**+**	**+**	**+**
25	IPOH-M	**-**	**+**	**+**	**-**	**-**	**-**	**+**	**-**	**+**	**+**	**+**	**+**	**+**	**+**
26	IPOH-S	**-**	**+**	**+**	**-**	**-**	**-**	**+**	**-**	**+**	**+**	**+**	**+**	**+**	**+**
27	IPOH-V	**-**	**+**	**+**	**-**	**-**	**-**	**+**	**-**	**+**	**+**	**+**	**+**	**+**	**+**
28	IPOH-Z	**-**	**+**	**+**	**-**	**-**	**-**	**+**	**-**	**+**	**+**	**+**	**+**	**+**	**+**

Abbreviation: NI^1^ = Number of strains; ID^2^ = Strains designation; GS^3^ = Gram-stain; KOH^4^ = Potassium hydroxide test; CR^5^ = Catalase reaction; OR^6^ = Oxidase reaction; IP^7^ = Indole production; M^8^ = Motility; SH^9^ = Starch hydrolysis; T80^10^ = Tween80 hydrolysis; GL^11^ = Gelatin liquefaction; PT^12^ = Potato Test; CT^13^ = Carbohydrate Test; G^14^ = Glucose; S^15^ = Sucrose; F^16^ = Fructose; L^17^ = Lactose.

Note: **+** = Positive reaction;**—** = Negative reaction.

### Hypersensitive assay

**Fig 4:**
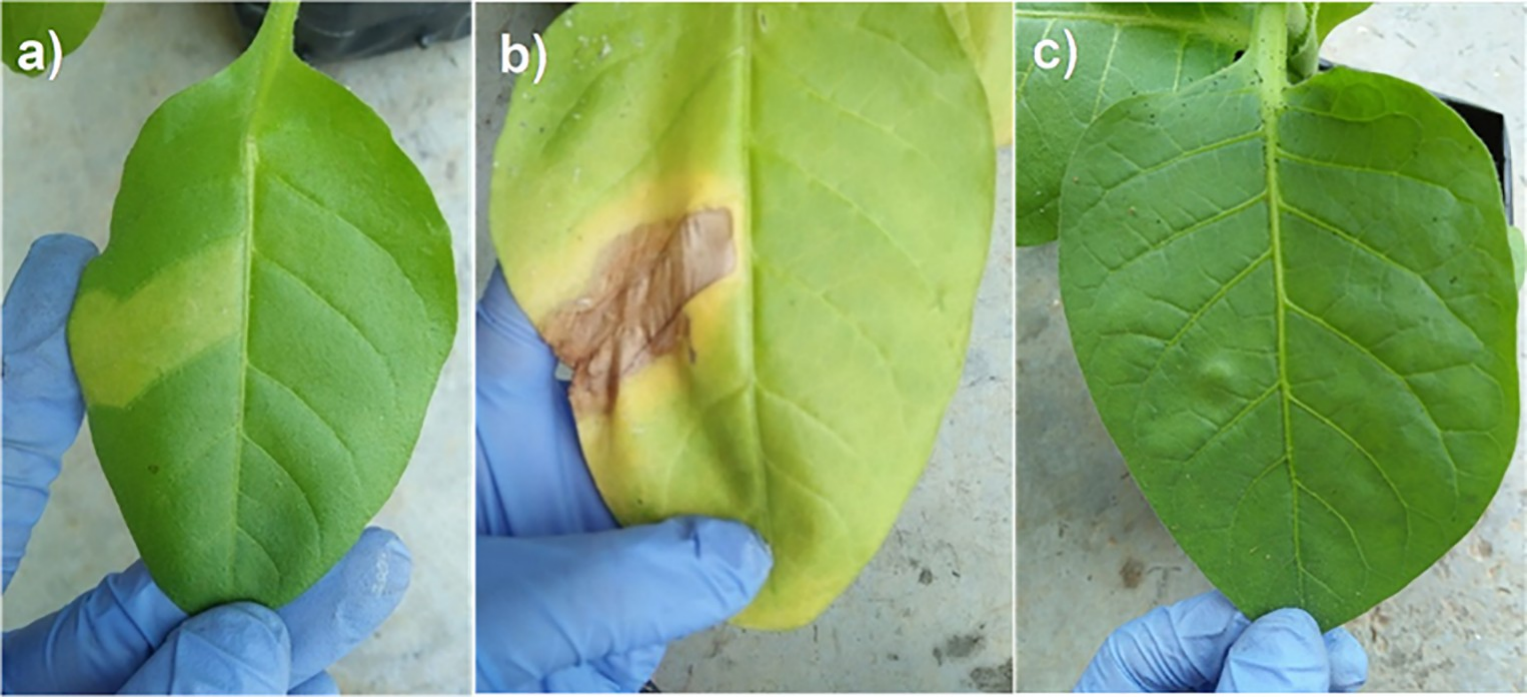
Tobacco infiltration assay of *Pantoea stewartii* subspecies *stewartii* strains (a) 12 h after infiltration tobacco leaf inoculated with JEN-14; and (b) 24 h after infiltration tobacco leaf inoculated with JEN-14; (c) Control tobacco plant. The hypersensitive reaction was induced by all *Pantoea stewartii* subsp. *stewartii* strains on tobacco plant under the greenhouse condition.

Hypersensitivity response (HR) induced by all 28 strains at 24 h after inoculation is shown in Fig 4. Necrotic lesions or chlorosis was observed up to 3 days post-infiltration. No changes appeared on the control tobacco plants.

### Pathogenicity test

**Fig 5:**
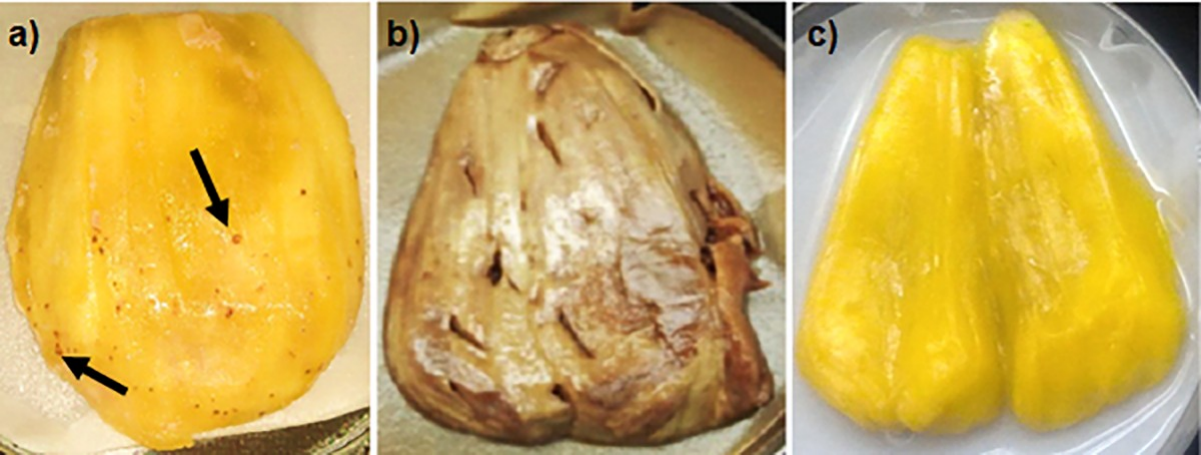
Pathogenicity of *Pantoea stewartii* subspecies *stewartii* strains on day 14 post-inoculation. (a) Yellowish discolouration with bronzing specks inoculated with JEN-14 strain; (b) Reddish discolouration inoculated with MAR-D strain; (c) Control jackfruit pulp.

All 28 *P*. *stewartii* subsp. *stewartii* strains produced jackfruit-bronzing disease symptoms within 14 days post-inoculation (Fig 5). No disease symptom appeared on the control. The re-isolation of the inoculated strains were tested further via phenotypic and molecular characterization and showed similar characteristics to positive *P*. *stewartii* subsp. *stewartii* test.

### MLSA- single and concatenated *gyr*B, *rpo*B, *atp*D and *inf*B gene sequences

**Fig 6:**
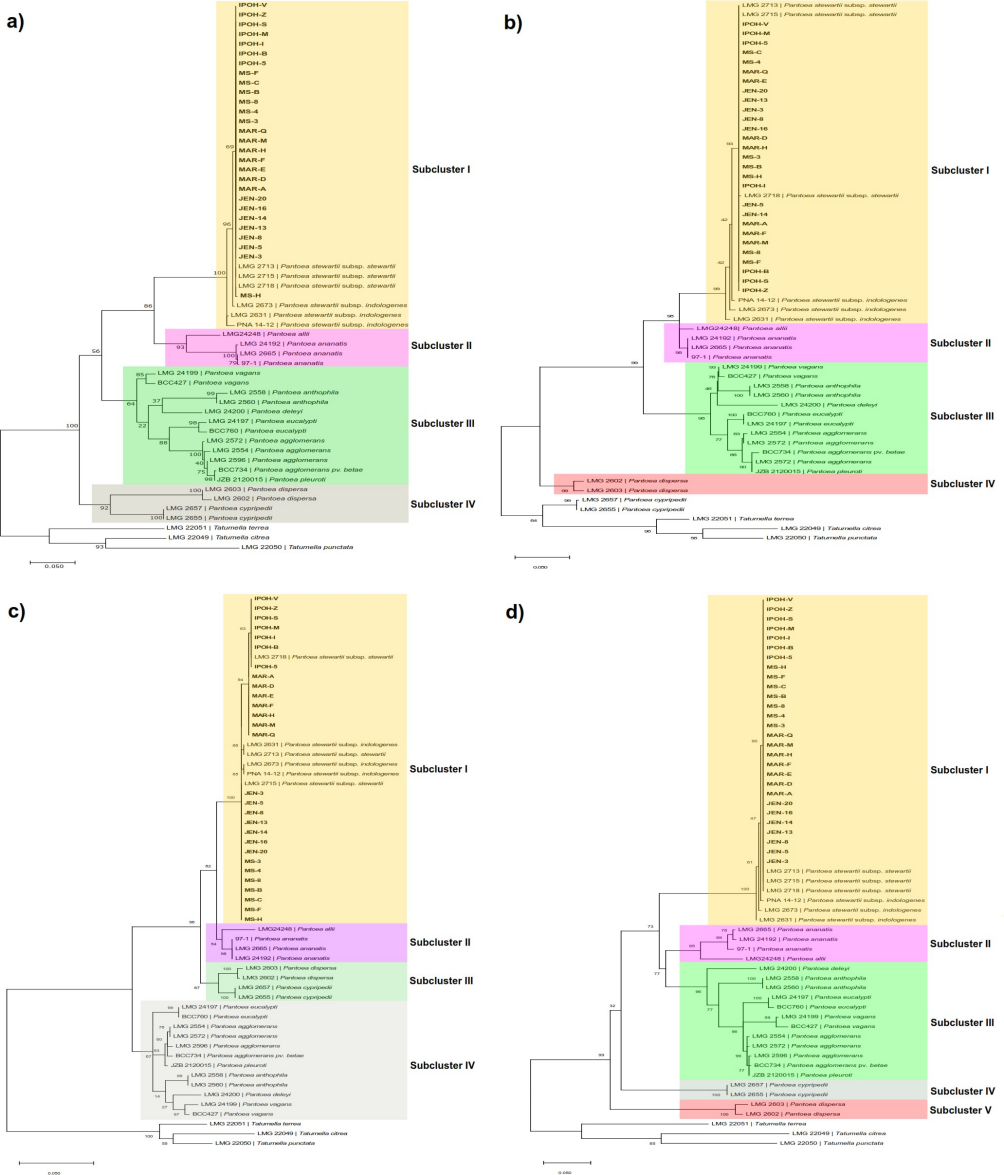
Maximum-likelihood tree based on the (a) *gyr*B; (b) *rpo*B; (c) *atp*D; and (d) *inf*B genes. Percentage bootstrap values based on 1000 replicates are given at nodes. *Tatumella* species were included as an outgroup. Bar: 0.05 substitutions per nucleotide position.

**Fig 7:**
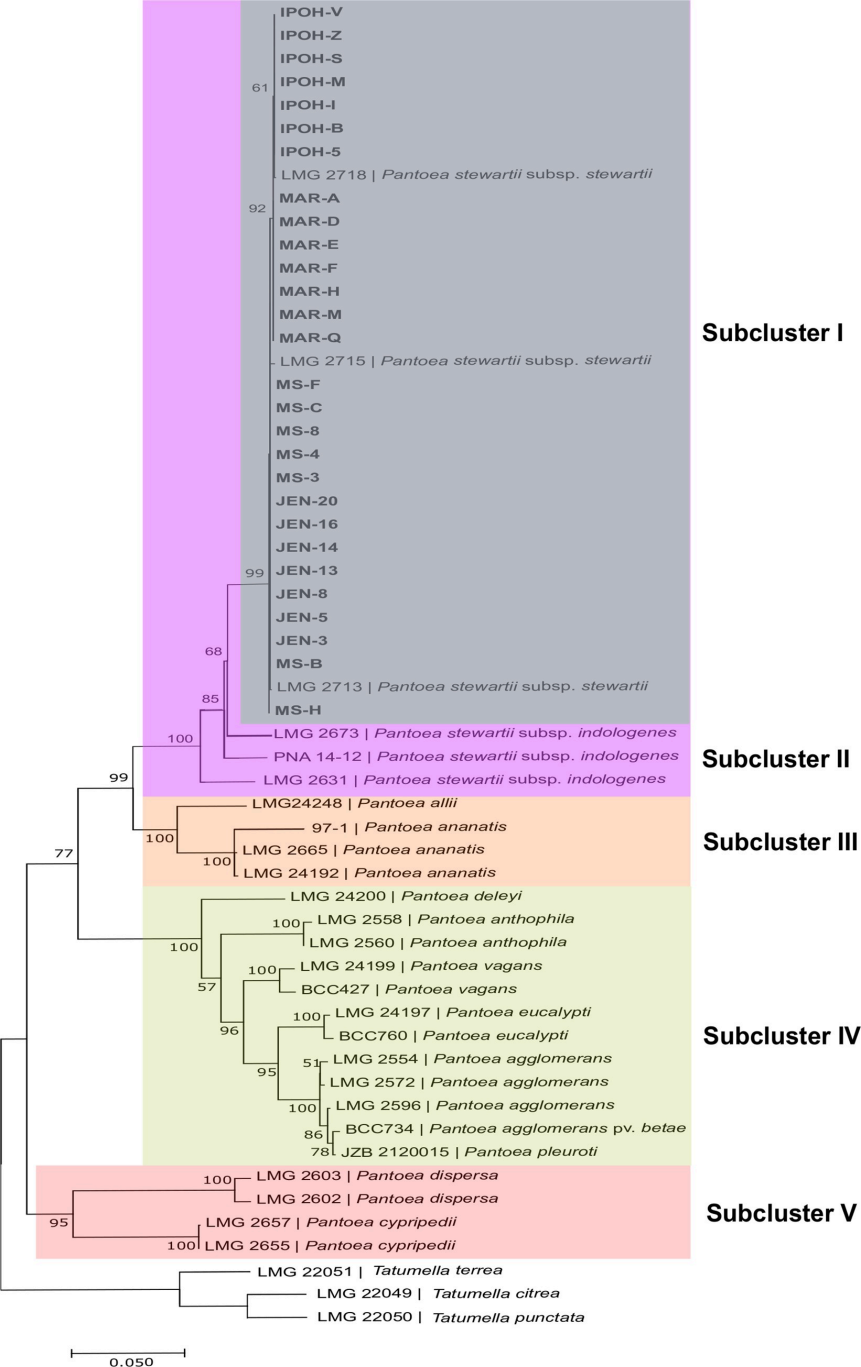
Maximum-likelihood tree based on the concatenated genes (*gyr*B, *rpo*B, *atp*D and *inf*B). Percentage bootstrap values based on 1000 replicates are given at nodes. *Tatumella* species were included as an outgroup. Bar: 0.05 substitutions per nucleotide position.

Phylogenetic trees computed based on single *gyr*B, *rpo*B, *atp*D and *inf*B genes (Fig 6), all revealed 99–100% similarities between our 28 bacterial strains with *P*. *stewartii* subsp. *stewartii* reference strains LMG 2713 (USA), LMG 2715 (USA), LMG 2718 (USA) and *P*. *stewartii* subsp. *indolegenes* reference strains PNA 14–12 (USA), LMG 2673 (Hawaii, USA) and LMG 2631 (India) reference strains (Sub-cluster I). Similarly, phylogenetic trees computed based on concatenated (Fig 7) from *gyr*B, *rpo*B, *atp*D and *inf*B genes showed 100% similarities between the 28 bacterial strains with *P*. *stewartii* subsp. *stewartii* reference strains LMG 2713 (USA), LMG 2715 (USA), LMG 2718 (USA) and *P*. *stewartii* subsp. *indolegenes* reference strains PNA 14–12 (USA), LMG 2673 (Hawaii, USA) and LMG 2631 (India) reference strains. However, although the sequence similarity between the 28 bacterial strains with subspecies *stewartii* and *indolegenes* were high (100% similarities) (Sub-cluster II), our strains were more closely related *to P*. *stewartii* subsp. *stewartii* (99% similarities) from its close relative *P*. *stewartii* subsp. *indologenes* (Sub-cluster I). All the strains collected from the four collection areas clustered together, showing no variation between the collection area of the bacterial strains.

## Discussion

Systematic identification and characterization were performed for the verification of *P*. *stewartii* subsp. *stewartii* strains from the 4 different jackfruit-bronzing disease outbreak collection area in Peninsular Malaysia. The strains were verified by morphological, phenotypic and molecular characterization test listed by (9). Starting with the isolation and phenotypic characterization, phenotypic tests were performed. Our study showed all 28 strains demonstrated a similar and parallel response to all phenotypic tests and resembled *P*. *stewartii* subsp. *stewartii* characteristics [5,9,17–19]. Motility test is crucial because stated that most bacteria under *Pantoea* genus are motile due to the presence of peritrichous flagella except for *P*. *stewartii* subsp. *stewartii* [11,23,24]. Indole test is also important in subspecies identification [18,25], as *P*. *stewartii* subsp. *indolegenes* distinguished and identified from *P*. *stewartii* subsp. *stewartii* through the positive result of indole test with Kovac’s reagent. So, the outcome of our indole and motility test results from the 28 strains showed strong positivity towards the identification of *P*. *stewartii* subsp. *stewartii*.

HR on tobacco (*Nicotiana tabacum*) was performed on *P*. *stewartii* subsp. *stewartii* and shown to be necrotic [14,18,26]. *P*. *stewartii* subsp. *stewartii* contains and carries a specialized protein secretion complex known as type III secretion systems (T3SSs) producing needle-like injectisomes for the delivery of effector proteins into host cells [11,27]. A previous study by Correa et al. (2012) confirmed that *P*. *stewartii* subsp. *stewartii* obtained from maize contains Hrc-Hrp1 as well as Inv-Mxi-Spa type T3SS, which were responsible for the pathogen colonization of susceptible hosts, and transmission from the vector to host plants by the transversal of the thick plant cell wall through the generation of thin pili. This *hrp* gene (resistant genes) secretion of *P*. *stewartii* subsp. *stewartii* is required to elicit a HR in tobacco leaves [14,28,29]. HR involves rapid reaction, and sometimes it only takes 8–12 h after being inoculated with the bacterial pathogen to become necrotic and eventually die [29]. Our hypersensitivity assay result was similar with previous reports [14,18], T3SSs and *hrp* genes were believed to promote parasitism and leads to pathogenesis, so other than virulence, tobacco hypersensitivity is also a very convenient way to screen bacterial cultures for pathogenicity [11,30].

Further confirmation was obtained from molecular characterization analysis. Standard differentiation of bacterial species using 16S-23S ITS region was believed to produce better sequence variability than the 16S rDNA gene as 16S rDNA gene cannot be separated easily by using electrophoresis [20]. Primer pair ES16/ESIG2c was tested on *P*. *stewartii* (33 strains), other *Pantoea* spp. (12 strains), related *Erwinia* spp. (14 strains) and *Xanthomonas campestris* pv. *campestris* (1 strain) and showed that only *P*. *stewartii* strains and one *P*. *ananas* (*P*. *ananatis)* strain yielded at the expected amplicons (0.92-kb fragment) [14]. This primer pair also confirmed the jackfruit-bronzing disease from Muadzam Shah plantation of Pahang state was caused by *P*. *stewartii* subsp. *stewartii* [6]. Our study has proven that all our 28 strains yielded at the expected amplicon’s size and sequence similarity of 100% to other *P*. *stewartii* subsp. *stewartii* strains obtained from GenBank database. Phylogenetic analysis constructed using MEGA7 on the ES16/ES1G2c gene sequences showed our strains clustered with the *P*. *stewartii* subsp. *stewartii* reference strains and none of the strains clustered to *P*. *agglomerans and P*. *ananatis* strains.

For the first time, genetic diversity of *P*. *stewartii* subsp. *stewartii* strains causing jackfruit-bronzing disease in Malaysia has been studied via MLSA. MLSA is a genotypic characterization method using the sequences of multiple protein-coding genes of prokaryotes for taxonomic purposes [31]. The stable housekeeping genes that codes for functional proteins make MLSA as a powerful method with a rapid genetic modification in comparison to 16S rDNA/rRNA [31]. Previous genetic diversity studies using 16S rDNA/rRNA reported the clustering and close relationship of *P*. *stewartii* subsp. *stewartii* with *P*. *ananatis* and *P*. *agglomerans* [32,33], *P*. *stewartii* with *P*. *ananatis* [34], *P*. *stewartii* subsp. *stewartii* with *Erwinia stewartii* [35] and *P*. *stewartii* subsp. *stewartii* with *P*. *ananatis*, *Pantoea deleyi*, *P*. *agglomerans*, *Pantoea vagans*, *Pantoea eucalypti* [36]. Undoubtedly, MLSA overpowers 16S rDNA/rRNA in taxanomic discrepancies in the identification purposes of *Pantoea* genus [37–39]. Four housekeeping genes were used in this study, as recommended by previous studies on MLSA [40–44]. All phylogenetic analyses in this study were performed with bootstrap replication of 1000, as it supports higher phylogenetic differentiation of *Pantoea* species [40,45]. The three outgroups were previously known as ‘Japanese species’ of *Pantoea*, namely *Pantoea citrea*, *Pantoea punctata* and *Pantoea terrea* [40]. However, MLSA indicated that the taxonomic position of these “Japanese species” were clustered together with *Tatumella ptyseos*, so they were then transferred to the genus *Tatumella*.

Phylogenetic trees obtained from MLSA based on sequences of *gyr*B, *rpo*B, *atp*D and *inf*B confirmed the allocation of all our strains to the genus *Pantoea*. Phylogenetic analysis showed that all of the 28 bacterial strains collected from the four collection areas were clustered together and phylogenetically most closely related (bootstrap value of 99–100%) to *P*. *stewartii* subsp. *stewartii* and *P*. *stewartii* subsp. *indolegenes* reference strains. Our result is congruent with the previous studies of MLSA performed on *Pantoea* species where phylogenetic trees based on housekeeping genes (*gyr*B, *rpo*B, *atp*D and *inf*B) revealed 99–100% similarities between *P*. *stewartii* subsp. *stewartii* and *P*. *stewartii* subsp. *indolegenes* [35,40,45–51].

However, MLSA based on the concatenated genes were able to distinguish that our strains were more closely related *to P*. *stewartii* subsp. *stewartii* (99% similarities) from its close relative *P*. *stewartii* subsp. *indologenes*, although sequence similarity between the subspecies was high (up to 100%). Study of MLSA that performed less than four housekeeping genes were insufficient and more bias, due to low-resolution power or genes impaired [44]. High discriminatory power is gained from MLSA from the concatenated genes than single gene [52]. In other words, the number of genes selection must be considered to achieve more discriminatory power [52].

All bacterial strains collected from the four areas of Malaysia were clustered together with the reference strains, indicating that the strains were genetically similar and share close phylogenetic relatedness with each other. This emphasizes that all our strains were likely originated and derived from a single emergence a long time ago [53]. Based on phylogenetic tree of the concatenated sequences of the four housekeeping genes (Fig 7), no major changes in the clustering pattern appeared since the position of the *P*. *stewartii* subsp. *indologenes* reference strains were still in one cluster (100% similarity) with our bacterial strains and *P*. *stewartii* subsp. *stewartii* reference strains. Hence, in our case, all the 28 *P*. *stewartii* subsp. *stewartii* bacterial strains (single and concatenated) might have a common origin with *P*. *stewartii* subsp. *stewartii* collected from USA (LMG 2713, LMG 2715 and LMG 2718) and *P*. *stewartii* subsp. *indologenes* bacterial strains collected from USA, Hawaii and India (PNA 14–12, LMG 2673 and LMG 2631).

In conclusion, our study verified and confirmed that bacteria causing jackfruit-bronzing in four areas of Malaysia is *P*. *stewartii* subsp. *stewartii*. We highlighted the genetic diversity of *P*. *stewartii* subsp. *stewartii* associated with jackfruit-bronzing in Malaysia, providing new insight on no genetic variation among the collected strains. Phylogenetic analyses indicated that our strains were closely related to *P*. *stewartii* subsp. *stewartii* strains (concatenated genes) and both *P*. *stewartii* subsp. *stewartii* and *P*. *stewartii* subsp. *indologenes* reference strains (single genes). As the improvement in jackfruit industries in Malaysia is vital, once jackfruit-bronzing disease is properly investigated, management options can be deployed to mitigate the disease impact. Controlling plant disease involves proper verification of the causal agent, where in this study, we provide information on the phenotypic tests, hypersensitivity test, pathogenicity test, molecular test, as well as genetic diversity evaluation through MLSA of *P*. *stewartii* subsp. *stewartii*. The output and data of this study will be useful as a proper reference and documentation in quarantine purposes and prevention of the spread of jackfruit-bronzing disease and its causal pathogen, *P*. *stewartii* subsp. *stewartii*.

## Supporting information

S1 Table The expected results of phenotypic tests *Pantoea stewartii* subspecies *stewartii* from previous studies. (DOCX)

S2 Table List of amplification primers used in this study. (DOCX)

S3 Table List of sequencing primers used in this study. (DOCX)

S4 Table Origin and characterization of *Pantoea stewartii* subspecies *stewartii* strains from Malaysia and reference strains for 16S-23S Internally Transcribed Spacer (ITS) Region in this study. (DOCX)

S5 Table Origin and characterization of *Pantoea stewartii* subspecies *stewartii* strains from Malaysia and reference strains for MLSA in this study. (DOCX)

S1 Fig Agarose gels showing PCR product from 16S-23S Internally Transcribed Spacer (ITS) region. Size of DNA ladder (M) used was 100bp (GeneDireX, Inc.) The amplification fragments were ~900 bp. (a) Strains from Jenderam; (b) Strains from Maran; (c) Strains from Ipoh; (d) Strains from Muadzam Shah; and -NC represent the Negative control.
(TIF)
