# Supplementary material for: Genetic diversity of Pantoea stewartii subspecies stewartii causing jackfruit-bronzing disease in Malaysia
Source: PLoS One. 2020 Jun 12;15(6):e0234350. doi: 10.1371/journal.pone.0234350 (PMC7292391; doi:10.1371/journal.pone.0234350)
Supplement: S1 Table — (DOCX) [file pone.0234350.s001.docx]

**S1 Table.**

| **Test** | **Result** | **Reference** |
| --- | --- | --- |
| Gram staining | Red-pink & Gram-negative (negative reaction) | [1,2] |
| KOH^1^ test | Formed thread-like slime (positive reaction) | [3] |
| Catalase reaction | Bubble formation (positive reaction) | [2] |
| Oxidase reaction | No deep blue colour (negative reaction) | [1] |
| Indole production | Remained yellow colour after the addition of Kovacs’ Reagent (negative reaction) | [1,4] |
| Motility | Non-motile (negative Brownian movement and growth confined to the stab line in SIM^2^ medium; positive reaction) | [1,2] |
| Starch hydrolysis | Utilized starch (positive reaction) | [2] |
| Tween80 hydrolysis | No opaque haloes around colonies (negative reaction) | [2] |
| Gelatin liquefaction | Hydrolysed gelatin (positive reaction) | [2] |
| Potato test | Lesion/pit, not soft rot (positive reaction) | [2] |
| Carbohydrate Test:  glucose, sucrose, lactose, fructose | All positive reaction  (Fastest --> Slowest colour change from purple to yellow when Bromocresol purple added). | [2,5] |

Abbreviation: KOH^1^= Potassium hydroxide; SIM^2^= Sugar, Indole, and Motility.

**References**

1. EPPO. PM 7/60 (2) *Pantoea stewartii* subsp. *stewartii*. EPPO Bull. 2016;46: 226–236. doi:10.1111/epp.12303

2. Gapasin RM, Garcia RP, Christine T, Cruz CS De, Borines LM. Fruit Bronzing : a New Disease Affecting Jackfruit Caused by *Pantoea stewartii* ( Smith ) Mergaert et al . Ann Trop Res. 2014;36: 17–31.

3. Rahma H, Sinaga MS, Surahman M. First Report of Stewart’s Wilt of Maize Caused by *Pantoea stewartii* subsp. *stewartii* In Bogor District, Indonesia. J Int Soc Southeast Asian Agric Sci. 2014;20: 131–141. Available: https://www.issaas.org/journal/v20/02/journal-issaas-v20n2-13-rahma_etal.pdf

4. Gehring I, Wensing A, Gernold M, Wiedemann W, Coplin DL, Geider K. Molecular differentiation of *Pantoea stewartii* subsp. *indologenes* from subspecies *stewartii* and identification of new isolates from maize seeds. J Appl Microbiol. 2014;116: 1553–1562. doi:10.1111/jam.12467

5. Orio AGA, Brücher E, Plazas MC, Sayago P, Guerra F, De Rossi R, et al. First Report of Stewart’s wilt of maize in Argentina Caused by *Pantoea stewartii*. Plant Dis. 2012;96: 1819–1819. doi:10.1094/PDIS-07-12-0668-PDN
