# Supplementary material for: Genetic diversity of Pantoea stewartii subspecies stewartii causing jackfruit-bronzing disease in Malaysia
Source: PLoS One. 2020 Jun 12;15(6):e0234350. doi: 10.1371/journal.pone.0234350 (PMC7292391; doi:10.1371/journal.pone.0234350)
Supplement: S2 Table — (DOCX) [file pone.0234350.s002.docx]

**S2 Table.**

| **Primers** | **Sequence (5’-3’)** | **Primer length (bp)** | **References** |
| --- | --- | --- | --- |
| *gyr*B 01-F | TAA RTT YGA YGA YAA CTC YTA YAA AGT | 27 | [1] |
| *gyr*B 02-R | CMC CYT CCA CCA RGT AMA GTT | 21 | [1] |
| *rpo*B CM7-F | AAC CAG TTC CGC GTT GGC CTG | 21 | [1] |
| *rpo*B CM31b-R | CCT GAA CAA CAC GCT CGG A | 19 | [1] |
| *atp*D 01-F | RTA ATY GGM GCS GTR GTN GAY GT | 23 | [1] |
| *atp*D 02-R | TCA TCC GCM GGW ACR TAW AYN GCC TG | 26 | [1] |
| *inf*B 01-F | ATY ATG GGH CAY GTH GAY CA | 20 | [1] |
| *inf*B 02-R | ACK GAG TAR TAA CGC AGA TCC A | 22 | [1] |

**References**

1. Brady C, Cleenwerck I, Venter S, Vancanneyt M, Swings J, Coutinho T. Phylogeny and identification of *Pantoea* species associated with plants, humans and the natural environment based on multilocus sequence analysis (MLSA). Syst Appl Microbiol. 2008;31: 447–460. doi:10.1016/j.syapm.2008.09.004
