# Supplementary material for: Genetic diversity of Pantoea stewartii subspecies stewartii causing jackfruit-bronzing disease in Malaysia
Source: PLoS One. 2020 Jun 12;15(6):e0234350. doi: 10.1371/journal.pone.0234350 (PMC7292391; doi:10.1371/journal.pone.0234350)
Supplement: S3 Table — (DOCX) [file pone.0234350.s003.docx]

**S3 Table.**

| **Primers** | **Sequence (5’-3’)** | **Primer length (bp)** | **References** |
| --- | --- | --- | --- |
| *gyr*B 07-F | GTV CGT TTC TGG CCV AG | 17 | [1] |
| *gyr*B 08-R | CTT TAC GRC GKG TCA TWT CAC | 21 | [1] |
| *rpo*B CM81-F | CAG TTC CGC GTT GGC CTG | 18 | [1] |
| *rpo*B CM81b-F | TGA TCA ACG CCA AGC C | 16 | [1] |
| *rpo*B CM32b-R | CGG ACC GGC CTG ACG TTG CAT | 21 | [1] |
| *atp*D 03-F | TGC TGG AAG TKC AGC ARC AG | 20 | [1] |
| *atp*D 04-R | CCM AGY ART GCG GAT ACT TC | 20 | [1] |
| *atp*D 08-R | CCG AGC AGC GCG GAG ATC TC | 20 | [2] |
| *inf*B 03-F | ACG GBA TGA TYA CST TCC TGG | 21 | [1] |
| *inf*B 04-R | AGY TTA GAT TTC TGC TGA CG | 20 | [1] |

**References**

1. Brady C, Cleenwerck I, Venter S, Vancanneyt M, Swings J, Coutinho T. Phylogeny and identification of *Pantoea* species associated with plants, humans and the natural environment based on multilocus sequence analysis (MLSA). Syst Appl Microbiol. 2008;31: 447–460. doi:10.1016/j.syapm.2008.09.004

2. Moretti C, Hosni T, Vandemeulebroecke K, Brady C, De Vos P, Buonaurio R, et al. *Erwinia oleae* sp. nov., isolated from olive knots caused by *Pseudomonas savastanoi* pv. *savastanoi*. Int J Syst Evol Microbiol. 2011;61: 2745–2752. doi:10.1099/ijs.0.026336-0
