# Supplementary material for: Genetic diversity of Pantoea stewartii subspecies stewartii causing jackfruit-bronzing disease in Malaysia
Source: PLoS One. 2020 Jun 12;15(6):e0234350. doi: 10.1371/journal.pone.0234350 (PMC7292391; doi:10.1371/journal.pone.0234350)
Supplement: S4 Table — (DOCX) [file pone.0234350.s004.docx]

**S4 Table.**

| Strains | Origin | Host | Species | GenBank accession | Reference |
| --- | --- | --- | --- | --- | --- |
| JEN-3 | Malaysia | *Artocarpus heterophyllus* | *Pantoea stewartii* subspecies *stewartii* | MK802515 | This study |
| JEN-5 | Malaysia | *Artocarpus heterophyllus* | *Pantoea stewartii* subspecies *stewartii* | MK802516 | This study |
| JEN-8 | Malaysia | *Artocarpus heterophyllus* | *Pantoea stewartii* subspecies *stewartii* | MK802517 | This study |
| JEN-13 | Malaysia | *Artocarpus heterophyllus* | *Pantoea stewartii* subspecies *stewartii* | MK802518 | This study |
| JEN-14 | Malaysia | *Artocarpus heterophyllus* | *Pantoea stewartii* subspecies *stewartii* | MK802519 | This study |
| JEN-16 | Malaysia | *Artocarpus heterophyllus* | *Pantoea stewartii* subspecies *stewartii* | MK802520 | This study |
| JEN-20 | Malaysia | *Artocarpus heterophyllus* | *Pantoea stewartii* subspecies *stewartii* | MK802521 | This study |
| MAR-A | Malaysia | *Artocarpus heterophyllus* | *Pantoea stewartii* subspecies *stewartii* | MK802522 | This study |
| MAR-D | Malaysia | *Artocarpus heterophyllus* | *Pantoea stewartii* subspecies *stewartii* | MK802523 | This study |
| MAR-E | Malaysia | *Artocarpus heterophyllus* | *Pantoea stewartii* subspecies *stewartii* | MK802524 | This study |
| MAR-F | Malaysia | *Artocarpus heterophyllus* | *Pantoea stewartii* subspecies *stewartii* | MK802525 | This study |
| MAR-H | Malaysia | *Artocarpus heterophyllus* | *Pantoea stewartii* subspecies *stewartii* | MK802526 | This study |
| MAR-M | Malaysia | *Artocarpus heterophyllus* | *Pantoea stewartii* subspecies *stewartii* | MK802527 | This study |
| MAR-Q | Malaysia | *Artocarpus heterophyllus* | *Pantoea stewartii* subspecies *stewartii* | MK802528 | This study |
| MS-3 | Malaysia | *Artocarpus heterophyllus* | *Pantoea stewartii* subspecies *stewartii* | MK802529 | This study |
| MS-4 | Malaysia | *Artocarpus heterophyllus* | *Pantoea stewartii* subspecies *stewartii* | MK802530 | This study |
| MS-8 | Malaysia | *Artocarpus heterophyllus* | *Pantoea stewartii* subspecies *stewartii* | MK802531 | This study |
| MS-B | Malaysia | *Artocarpus heterophyllus* | *Pantoea stewartii* subspecies *stewartii* | MK802532 | This study |
| MS-C | Malaysia | *Artocarpus heterophyllus* | *Pantoea stewartii* subspecies *stewartii* | MK802533 | This study |
| MS-F | Malaysia | *Artocarpus heterophyllus* | *Pantoea stewartii* subspecies *stewartii* | MK802534 | This study |
| MS-H | Malaysia | *Artocarpus heterophyllus* | *Pantoea stewartii* subspecies *stewartii* | MK802535 | This study |
| IPOH-5 | Malaysia | *Artocarpus heterophyllus* | *Pantoea stewartii* subspecies *stewartii* | MK531591 | This study |
| IPOH-B | Malaysia | *Artocarpus heterophyllus* | *Pantoea stewartii* subspecies *stewartii* | MK802536 | This study |
| IPOH-I | Malaysia | *Artocarpus heterophyllus* | *Pantoea stewartii* subspecies *stewartii* | MK802537 | This study |
| IPOH-M | Malaysia | *Artocarpus heterophyllus* | *Pantoea stewartii* subspecies *stewartii* | MK802538 | This study |
| IPOH-S | Malaysia | *Artocarpus heterophyllus* | *Pantoea stewartii* subspecies *stewartii* | MK802539 | This study |
| IPOH-V | Malaysia | *Artocarpus heterophyllus* | *Pantoea stewartii* subspecies *stewartii* | MK802540 | This study |
| IPOH-Z | Malaysia | *Artocarpus heterophyllus* | *Pantoea stewartii* subspecies *stewartii* | MK802541 | This study |
| DC283 | USA | Corn | *Pantoea stewartii* subspecies *stewartii* | AJ311838 | [1] |
| S3 | Malaysia | *Artocarpus heterophyllus* | *Pantoea stewartii* subspecies *stewartii* | KY195918 | [2] |
| W1 | Malaysia | *Artocarpus heterophyllus* | *Pantoea stewartii* subspecies *stewartii* | MF598163 | [3] |
| Hcar02a | Tunisia | *Hedysarum carnosum* | *Pantoea agglomerans* | JQ081302 | [4] |
| AJ13355 | Japan | Soil | *Pantoea ananatis* | NR076293 | [5] |

References

1. Coplin DL, Majerczak DR, Zhang Y, Kim W-S, Jock S, Geider K. Identification of *Pantoea stewartii* subsp. *stewartii* by PCR and strain differentiation by PFGE. 2002. Available: https://apsjournals.apsnet.org/doi/pdf/10.1094/PDIS.2002.86.3.304

2. Zulperi D, Manaf N, Ismail SI, Karam DS, Yusof MT. First Report of *Pantoea stewartii* subsp. *stewartii* Causing Fruit Bronzing of Jackfruit (*Artocarpus heterophyllus*), a New Emerging Disease in Peninsular Malaysia. Plant Dis. 2017;101: 831–831. doi:10.1094/PDIS-11-16-1689-PDN

3. Ismail-Suhaimy NW, Zulperi D, Tan SQ, Ismail SI, Karam DS, Yusof MT, et al. Characterization of *Pantoea stewartii* subspecies *stewartii* causing bronzing disease of jackfruit in Malaysia. Unpubl J. 2017. Available: https://www.ncbi.nlm.nih.gov/nuccore/1409964353

4. Chriki R, Chriki A. Phylogenetic Study of Rhizobia isolated from root-nodules of Hedysarum legumes. Unpubl J. 2012. Available: https://www.ncbi.nlm.nih.gov/nuccore/JQ081302

5. Hara Y. Genome sequence of Pantoea ananatis AJ13355. Unpubl J. 2010.
