# Supplementary material for: Genetic diversity of Pantoea stewartii subspecies stewartii causing jackfruit-bronzing disease in Malaysia
Source: PLoS One. 2020 Jun 12;15(6):e0234350. doi: 10.1371/journal.pone.0234350 (PMC7292391; doi:10.1371/journal.pone.0234350)
Supplement: S5 Table — (DOCX) [file pone.0234350.s005.docx]

**S5 Table.**

| **Strains** | **Origin** | **Host** | **Species** | **GenBank accession** | | | | **Reference** |
| --- | --- | --- | --- | --- | --- | --- | --- | --- |
|  |  |  |  | ***gyr*B** | ***rpo*B** | ***atp*D** | ***inf*B** |  |
| JEN-3 | Malaysia | *Artocarpus heterophyllus* | *Pantoea stewartii* subspecies *stewartii* | MK791376 | MK820908 | MK791348 | MK791320 | This study |
| JEN-5 | Malaysia | *Artocarpus heterophyllus* | *Pantoea stewartii* subspecies *stewartii* | MK791377 | MK820909 | MK791349 | MK791321 | This study |
| JEN-8 | Malaysia | *Artocarpus heterophyllus* | *Pantoea stewartii* subspecies *stewartii* | MK791378 | MK820910 | MK791350 | MK791322 | This study |
| JEN-13 | Malaysia | *Artocarpus heterophyllus* | *Pantoea stewartii* subspecies *stewartii* | MK791379 | MK820911 | MK791351 | MK791323 | This study |
| JEN-14 | Malaysia | *Artocarpus heterophyllus* | *Pantoea stewartii* subspecies *stewartii* | MK791380 | MK820912 | MK791352 | MK791324 | This study |
| JEN-16 | Malaysia | *Artocarpus heterophyllus* | *Pantoea stewartii* subspecies *stewartii* | MK791381 | MK820913 | MK791353 | MK791325 | This study |
| JEN-20 | Malaysia | *Artocarpus heterophyllus* | *Pantoea stewartii* subspecies *stewartii* | MK791382 | MK820914 | MK791354 | MK791326 | This study |
| MAR-A | Malaysia | *Artocarpus heterophyllus* | *Pantoea stewartii* subspecies *stewartii* | MK791383 | MK820915 | MK791355 | MK791327 | This study |
| MAR-D | Malaysia | *Artocarpus heterophyllus* | *Pantoea stewartii* subspecies *stewartii* | MK791384 | MK820916 | MK791356 | MK791328 | This study |
| MAR-E | Malaysia | *Artocarpus heterophyllus* | *Pantoea stewartii* subspecies *stewartii* | MK791385 | MK820917 | MK791357 | MK791329 | This study |
| MAR-F | Malaysia | *Artocarpus heterophyllus* | *Pantoea stewartii* subspecies *stewartii* | MK791386 | MK820918 | MK791358 | MK791330 | This study |
| MAR-H | Malaysia | *Artocarpus heterophyllus* | *Pantoea stewartii* subspecies *stewartii* | MK791387 | MK820919 | MK791359 | MK791331 | This study |
| MAR-M | Malaysia | *Artocarpus heterophyllus* | *Pantoea stewartii* subspecies *stewartii* | MK791388 | MK820920 | MK791360 | MK791332 | This study |
| MAR-Q | Malaysia | *Artocarpus heterophyllus* | *Pantoea stewartii* subspecies *stewartii* | MK791389 | MK820921 | MK791361 | MK791333 | This study |
| MS-3 | Malaysia | *Artocarpus heterophyllus* | *Pantoea stewartii* subspecies *stewartii* | MK791390 | MK820922 | MK791362 | MK791334 | This study |
| MS-4 | Malaysia | *Artocarpus heterophyllus* | *Pantoea stewartii* subspecies *stewartii* | MK791391 | MK820923 | MK791363 | MK791335 | This study |
| MS-8 | Malaysia | *Artocarpus heterophyllus* | *Pantoea stewartii* subspecies *stewartii* | MK791392 | MK820924 | MK791364 | MK791336 | This study |
| MS-B | Malaysia | *Artocarpus heterophyllus* | *Pantoea stewartii* subspecies *stewartii* | MK791393 | MK820925 | MK791365 | MK791337 | This study |
| MS-C | Malaysia | *Artocarpus heterophyllus* | *Pantoea stewartii* subspecies *stewartii* | MK791394 | MK820926 | MK791366 | MK791338 | This study |
| MS-F | Malaysia | *Artocarpus heterophyllus* | *Pantoea stewartii* subspecies *stewartii* | MK791395 | MK820927 | MK791367 | MK791339 | This study |
| MS-H | Malaysia | *Artocarpus heterophyllus* | *Pantoea stewartii* subspecies *stewartii* | MK791396 | MK820928 | MK791368 | MK791340 | This study |
| IPOH-5 | Malaysia | *Artocarpus heterophyllus* | *Pantoea stewartii* subspecies *stewartii* | MK791397 | MK820929 | MK791369 | MK791341 | This study |
| IPOH-B | Malaysia | *Artocarpus heterophyllus* | *Pantoea stewartii* subspecies *stewartii* | MK791398 | MK820930 | MK791370 | MK791342 | This study |
| IPOH-I | Malaysia | *Artocarpus heterophyllus* | *Pantoea stewartii* subspecies *stewartii* | MK791399 | MK820931 | MK791371 | MK791343 | This study |
| IPOH-M | Malaysia | *Artocarpus heterophyllus* | *Pantoea stewartii* subspecies *stewartii* | MK791400 | MK820932 | MK791372 | MK791344 | This study |
| IPOH-S | Malaysia | *Artocarpus heterophyllus* | *Pantoea stewartii* subspecies *stewartii* | MK791401 | MK820933 | MK791373 | MK791345 | This study |
| IPOH-V | Malaysia | *Artocarpus heterophyllus* | *Pantoea stewartii* subspecies *stewartii* | MK791402 | MK820934 | MK791374 | MK791346 | This study |
| IPOH-Z | Malaysia | *Artocarpus heterophyllus* | *Pantoea stewartii* subspecies *stewartii* | MK791403 | MK820935 | MK791375 | MK791347 | This study |
| LMG 2713 | USA | Corn | *Pantoea stewartii* subspecies *stewartii* | EF988830 | EF989002 | EF988743 | EF988916 | [1] |
| LMG 2715 | USA | Corn | *Pantoea stewartii* subspecies *stewartii* | EF988831 | EF989003 | EF988744 | EF988917 | [1] |
| LMG 2718 | USA | Corn | *Pantoea stewartii* subspecies *stewartii* | EF988832 | EF989004 | EF988745 | EF988918 | [1] |
| LMG 2631 | India | Fox Millet | *Pantoea stewartii* subspecies *indolegenes* | EF988822 | EF988994 | EF988735 | EF988908 | [1] |
| LMG 2673 | Hawaii, USA | Pineapple | *Pantoea stewartii* subspecies *indolegenes* | EF988827 | EF988999 | EF988740 | EF988914 | [1] |
| PNA 14-12 | USA | Onion | *Pantoea stewartii* subspecies *indolegenes* | MF771257 | MF771259 | MF771256 | MF771258 | [1] |
| LMG 2554 | UK | Scarlet runner bean | *Pantoea agglomerans* | EF988811 | EF988983 | EF988724 | EF988897 | [1] |
| LMG 2572 | Canada | Wheat | *Pantoea agglomerans* | EF988815 | EF988987 | EF988728 | EF988901 | [1] |
| LMG 2596 | South Africa | Onion | *Pantoea agglomerans* | EF988816 | EF988988 | EF988729 | EF988902 | [1] |
| BCC734 | Unknown | Beet | *Pantoea agglomerans* pv. *betae* | EF988774 | EF988946 | EF988687 | EF988860 | [1] |
| LMG 2665 | Brazil | Pineapple | *Pantoea ananatis* | EF988824 | EF988996 | EF988737 | EF988910 | [1] |
| LMG 24192 | South Africa | Maize | *Pantoea ananatis* | EF988785 | EF988957 | EF988698 | EF988871 | [1] |
| PNA 97-1 | USA | Onion | *Pantoea ananatis* | MF771246 | MF771248 | MF771245 | MF771247 | [2] |
| LMG 2602 | India | Sorghum | *Pantoea dispersa* | EF988817 | EF988989 | EF988730 | EF988903 | [1] |
| LMG 2603 | Japan | Soil | *Pantoea dispersa* | EF988818 | EF988990 | EF988731 | EF988904 | [1] |
| LMG 2558 | India | *Impatiens balsamina* | *Pantoea anthophila* | EF988812 | EF988984 | EF988725 | EF988898 | [1] |
| LMG 2560 | Unknown | *Tagetes erecta* | *Pantoea anthophila* | EF988813 | EF988985 | EF988726 | EF988899 | [1] |
| LMG 24197 | Eucalyptus | Uruguay | *Pantoea eucalypti* | EF988762 | EF988934 | EF988675 | EF988848 | [1] |
| BCC760 | Uraguay | Eucalyptus | *Pantoea eucalypti* | EF988777 | EF988949 | EF988690 | EF988863 | [1] |
| LMG 24248 | South Africa | Onion | *Pantoea allii* | EF988696 | EF988783 | EF988869 | EF988955 | [1] |
| LMG 24199 | Uganda | Eucalyptus | *Pantoea vagans* | EF988768 | EF988940 | EF988715 | EF988854 | [1] |
| BCC427 | Uganda | Eucalyptus | *Pantoea vagans* | EF988836 | EF989008 | EF988749 | EF988922 | [1] |
| LMG 2657 | USA | Orchid | *Pantoea cypripedii* | FJ187830 | FJ187840 | FJ187825 | FJ187835 | [1] |
| LMG 2655 | USA | Orchid | *Pantoea cypripedii* | FJ187831 | FJ187841 | FJ187826 | FJ187836 | [1] |
| LMG 24200 | Uganda | Eucalyptus | *Pantoea deleyi* | EF988770 | EF988942 | EF988683 | EF988856 | [1] |
| JZB 2120015 | China | *Pleurotus eryngii* | *Pantoea pleuroti* | KJ654342 | KJ654343 | KJ654344 | KJ654345 | [3] |
| LMG 22049 | Japan | Mandarin Orange | *Tatumella citrea* | EF988802 | EF988974 | EF988715 | EF988888 | [1] |
| LMG 22051 | Japan | Soil | *Tatumella terrea* | EF988804 | EF988976 | EF988717 | EF988890 | [1] |
| LMG 22050 | Japan | Mandarin Orange | *Tatumella punctata* | EF988803 | EF988975 | EF988716 | EF988889 | [1] |

**References**

1. Brady C, Cleenwerck I, Venter S, Vancanneyt M, Swings J, Coutinho T. Phylogeny and identification of *Pantoea* species associated with plants, humans and the natural environment based on multilocus sequence analysis (MLSA). Syst Appl Microbiol. 2008;31: 447–460. doi:10.1016/j.syapm.2008.09.004

2. Stice SP, Stumpf SD, Gitaitis RD, Kvitko BH, Dutta B. *Pantoea ananatis* Genetic Diversity Analysis Reveals Limited Genomic Diversity as Well as Accessory Genes Correlated with Onion Pathogenicity. Front Microbiol. 2018;9: 184. doi:10.3389/fmicb.2018.00184

3. Ma Y, Yin Y, Rong C, Chen S, Liu Y, Wang S, et al. *Pantoea pleuroti* sp. nov., Isolated from the Fruiting Bodies of *Pleurotus eryngii*. Curr Microbiol. 2016;72: 207–212. doi:10.1007/s00284-015-0940-5
