# Supplementary figures and images for: Genetic diversity of Pantoea stewartii subspecies stewartii causing jackfruit-bronzing disease in Malaysia
Source: PLoS One. 2020 Jun 12;15(6):e0234350. doi: 10.1371/journal.pone.0234350 (PMC7292391; doi:10.1371/journal.pone.0234350)

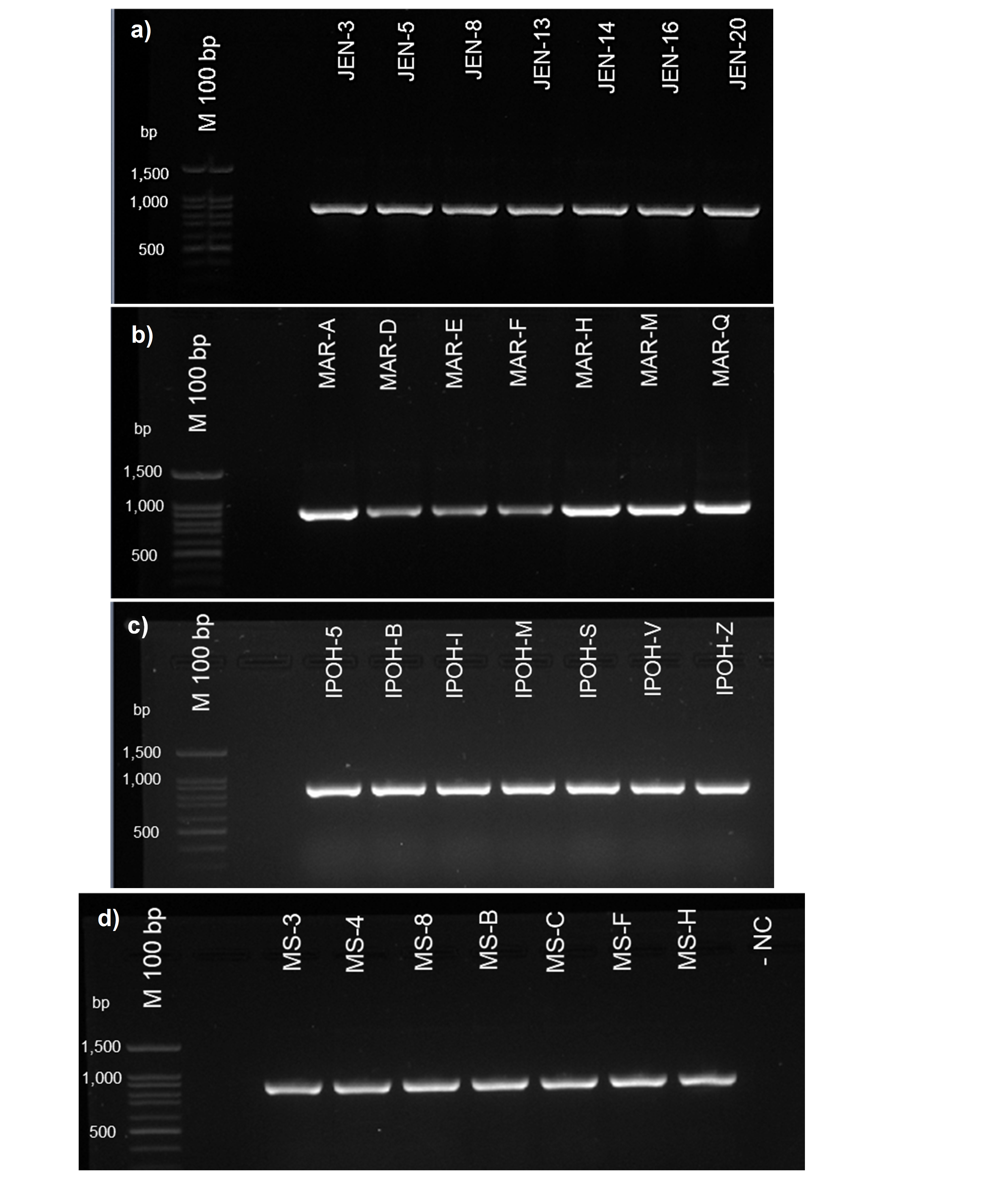

Supplement: S1 Fig — Size of DNA ladder (M) used was 100bp (GeneDireX, Inc.) The amplification fragments were ~900 bp. (a) Strains from Jenderam; (b) Strains from Maran; (c) Strains from Ipoh; (d) Strains from Muadzam Shah; and -NC represent the Negative control. (TIF) [file pone.0234350.s006.tif]
